# Research trends and hotspot analysis of age-related hearing loss from a bibliographic perspective

**DOI:** 10.3389/fpsyg.2022.921117

**Published:** 2022-09-22

**Authors:** Qingjia Cui, Na Chen, Cheng Wen, Jianing Xi, Lihui Huang

**Affiliations:** ^1^Rehabilitation Centre of Otolaryngology-Head and Neck, Beijing Rehabilitation Hospital, Capital Medical University, Beijing, China; ^2^Department of Otolaryngology-Head and Neck Surgery, Beijing Tongren Hospital, Capital Medical University, Beijing, China; ^3^Key Laboratory of Otolaryngology-Head and Neck Surgery, Ministry of Education, Beijing Institute of Otolaryngology, Beijing, China; ^4^Beijing Rehabilitation Hospital, Capital Medical University, Beijing, China

**Keywords:** age-related hearing loss, hotspot, research trends, visualization analysis, association

## Abstract

**Background:**

Up-to-date information about the trends of age-related hearing loss (ARHL) and how this varies between countries is essential to plan for an adequate health-system response. Therefore, this study aimed to assess the research hotpots and trends in ARHL and to provide the basis and direction for future research.

**Materials and methods:**

The Web of Science Core Collection database was searched and screened according to the inclusion criteria during 2002–2021. Bibliometric analyses were conducted by CiteSpace (Chaomei Chen, Drexel University, Philadelphia, PA, United States) software and VOSviewer (Center for Science and Technology Studies, Leiden University, Leiden, The Netherlands) software.

**Results:**

The query identified 1,496 publications, which showed a growth trend of this filed. These publications were from 62 countries, the United States of America (United States) showed its tremendous impact on this field in publication outputs, total citations, and international collaborations, China following in second. The *Journal of Hearing Research* was the most productive journal. Weijia Kong published the most papers, and the most productive institution was Washington University. The keyword “presbycusis” ranked first in research frontiers and appeared earlier, and the keywords “age-related hearing loss,” “risk,” “dementia,” “auditory cortex,” “association,” and “decline” began to appear in recent years.

**Conclusion:**

The annual number of publications has grown rapidly in the past two decades and will continue to grow. Epidemiological investigation and laboratory research are lasting hot spots, besides future research will focus on the association between ARHL and cognitive decline, dementia, and Alzheimer’s disease.

## Introduction

Age related hearing loss (ARHL) or presbycusis can be defined as a progressive, bilateral, and symmetrical sensorineural hearing loss due to age-related degeneration of inner ear structures (Bowl and Dawson, 2019). ARHL usually affects the high frequencies of hearing, and no recovery or incomplete revitalization, no history of noise contact, and the speech discrimination rate are not directly proportional to the pure tone hearing threshold (Bowl and Dawson, 2019). The resulting poor speech recognition in ARHL has a negative impact on cognitive, emotional, and social function in older adults (Christensen et al., 2019; Sardone et al., 2020). Globally, the prevalence of moderate or higher-grade severity hearing loss increases exponentially with age, increasing from 15.4% among people in their 60s to 58.2% among those aged >90 years (Homans et al., 2017). Current estimates suggest that >42% of people with any degree of hearing loss are older than 60 years of age (Quaranta et al., 2015). The World Health Organization reported a prevalence of ARHL across regions of 10.9–17.6% among individuals aged 60–69 years, increasing to 41.9–51.2% among those aged 80–89 years and reaching 52.9–64.9% in those older than 90 years of age (Chadha et al., 2021). Moreover, ARHL is the third top leading cause of years lived with disability diseases worldwide, followed low back pain and migraine, affecting 6–8% of the world’s population (GBD, 2018).

Considering the expected increases in life expectancy, the Global Burden of Disease estimated that the annual cost related to ARHL will increase to $60 billion by 2030 (Huddle et al., 2017). The health, societal and economic costs of ARHL are vast and ever-increasing. The global health systems will face increasing demand for services that are costlier than the interventions that have led to declines in mortality in childhood or for the major causes of mortality in adults (Brown et al., 2018). Therefore, the study of ARHL has very important social and practical importance. Up-to-date information about the trends of ARHL and how it varies between countries is essential to plan for an adequate health-system response.

Bibliometric analysis is a statistics method that summarizes and analyzes research papers and academic journals based on the public literature database such as Web of Science (Tran et al., 2018). Bibliometric analysis not only provides quantitative and qualitative evaluation of the publication, but also presents the most influential research quickly and accurately, which provides theoretical basis for further research (Zhang et al., 2020). CiteSpace and VOSviewer software use information from the Web of Science Core Collection and other database networks to evaluate the corresponding data around knowledge gap involving important information including authors, journals, research institutions and keywords, and has been widely used in many fields such as medicine, geology, and ecology (Gao et al., 2019; Zhou et al., 2021). This study aimed to assess the research hotpots and trends in ARHL and to provide the basis and direction for future research.

## Materials and methods

### Ethics statement

Ethical approval was not applicable in the present study.

### Data sources and search strategy

Relevant literature was searched from the Web of Science Core Collection (WoSCC) on July 24, 2022. With the availability of many bibliometric indicators and containing 12,000 influential high-quality journals from countries worldwide, the WoS database is regarded as one of the most authoritative and optimal databases for bibliometrics analysis of scientific publications. Apart from the general literature search, it also possesses an important function of citation index searching, which is helpful for assessing the academic performance of literature in a specific field (Wu et al., 2021a). The search strategy was as follows: TS = (“age-related hearing loss” OR “age-related hearing Impairment*” OR “age-related hearing disorder*” OR “age-related deafness” OR “old* adults hearing loss” OR “old* adults hearing Impairment*"OR “old* adults hearing disorder*” OR “old* adults deafness” OR presbycusis OR presbyacusis).

### Screening criteria

**Figure 1:**
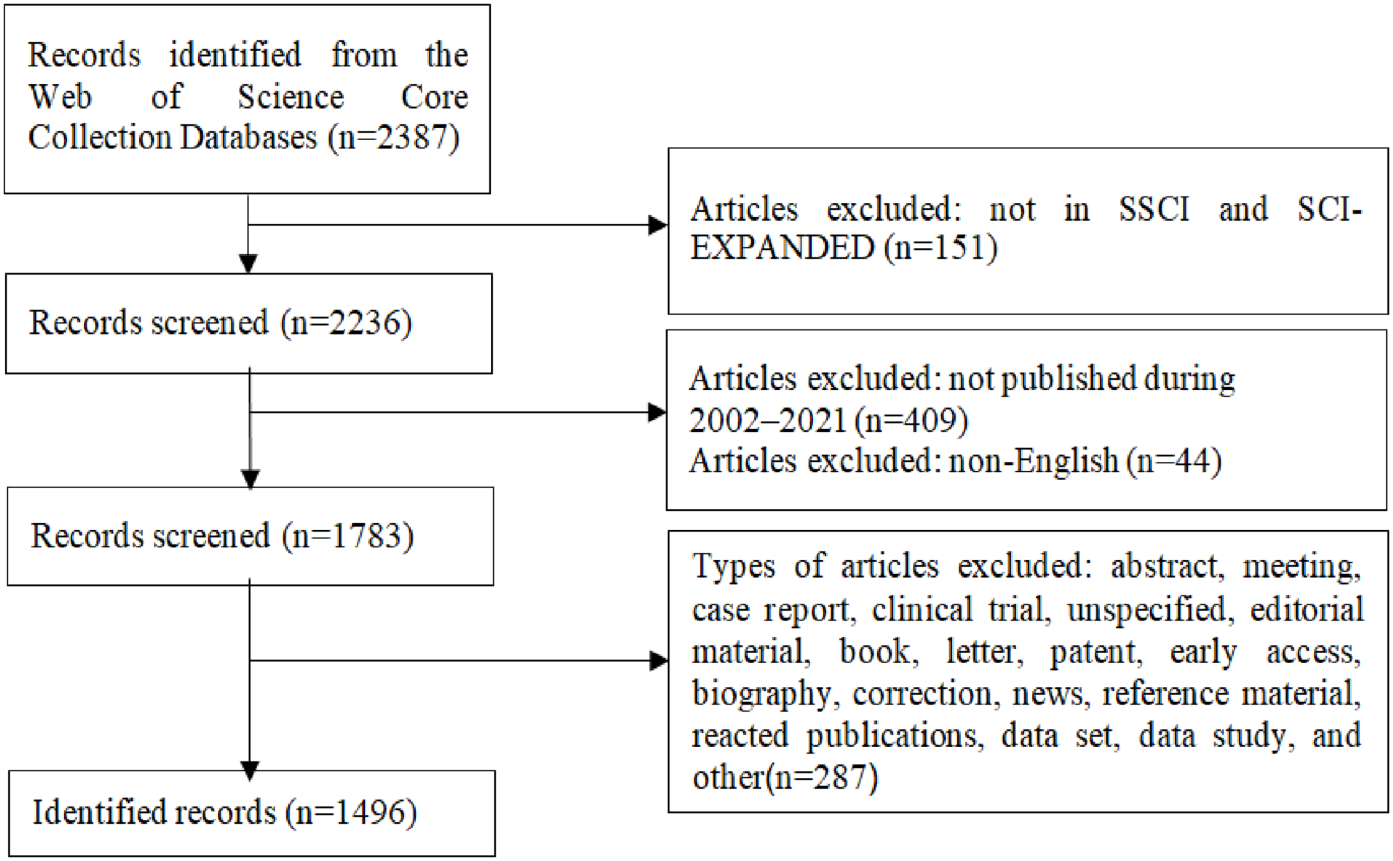
Flowchart of the included and excluded publications. SCCI, Social Sciences Citation Index; SCI-EXPANDED, Science Citation Index-Expanded.

The screening criteria are shown in Figure 1. After the initial search, we selected two authors to review and screen the initially searched publications based on the following inclusion criteria: (1) the language of publication is “English”; (2) the publication type is “article.” Reviews, editorials, letters to the editor, case reports retracted publications, book chapter, and meeting abstract were excluded; (3) the publication came from SCI-EXPANDED and SSCI; (4) the timespan is between 2002 and 2021; and (5) regarding the research content of the selected publication, the study population comprised patients with ARHL, including preoperative and postoperative ones, included an animal model of ARHL, or included a cell model of ARHL. According to the inclusion criteria, 1,496 publications were finally included in our study.

### Statistical analysis

The full record and all cited references of the included publications were downloaded and exported to CiteSpace (5.8.R3 [64-bit]; Chaomei Chen) and VOSviewer (1.6.18; enter for Science and Technology Studies) for further analysis. Analysis of CiteSpace included annual publications, centrality value of authors, countries and regions, and institutions, the overlay of the journals and cited journals, the cluster network graph and the timeline view of co-cited references, and keywords with the strongest citation bursts. VOSviewer 1.6.18 was used to analyze scientifically-based knowledge networks, including publication countries, institutions, clusters of journals and keywords. Microsoft Excel 2010 (Microsoft Corporation) was used to construct a polynomial regression model to predict the number of articles related to ARHL published in 2022.

## Results

### Global publications

**Figure 2:**
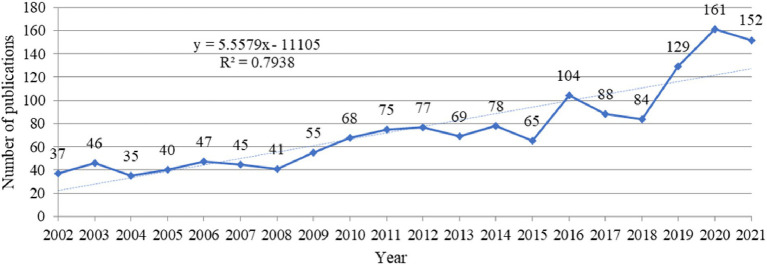
Annual number of globally published publications in the field of age-related hearing loss from 2002 to 2021.

In total, 1,496 publications on ARHL were published from 2002 to 2021. The annual number of articles published in each field show the development trend, as shown in Figure 2, which was created by inputted data in Excel. As shown in Figure 2, the time trend on research about ARHL comprised three phases: the first stage (2002–2008), the number of studies in the field was small and tended to be stable at an average of 30 per year; second stage (2009–2015), with the continuous increase of research published on ARHL by researchers, the number of papers published increased with little fluctuation; and third stage (2016 to 2021), since 2016, the number of publications increased considerably compared to the years before. A statistically significant relationship could be observed between publication year and the number of publications (*R*^2^ = 0.7938). A preliminary conclusion was drawn from the data from the recent 20 years: research on ARHL has grown in general, especially in the last 6 years.

### Contributions of the top 10 authors

**Figure 3:**
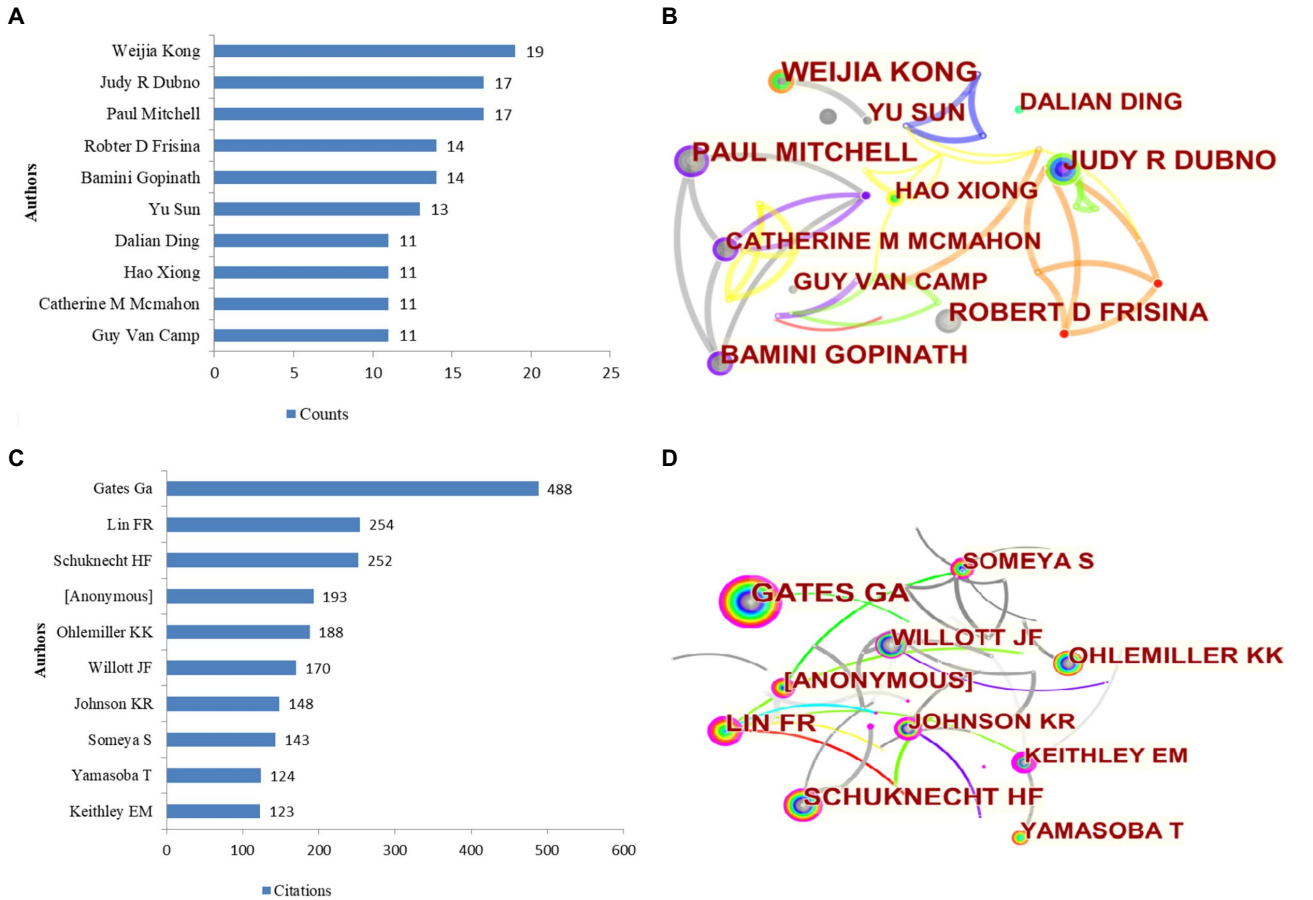
Top 10 authors in the field of age-related hearing loss from 2002 to 2021 **(A)** and a network map of top 10 authors **(B)**. Top 10 co-cited authors **(C)** and a network map of top 10 co-cited authors **(D)**.

Since 2002, a total of 5,514 authors have participated in publishing articles related to ARHL. According to CiteSpace, the top 10 most active authors are shown in Figure 3A. Figure 3B shows the authors collaboration in a network map. The size of the circle reflects the number of articles published by authors, and the lines connecting the circles represent co-occurrence relationships between authors. The distance between two authors indicates the relatedness of their co-authorship link, and a smaller distance implies a stronger correlation of their relatedness. And authors who are jointly cited are likely to focus on similar research areas. The representative author in the field of ARHL was Weijia Kong with a total of 19 publications, followed by Judy R Dubno (*n* = 17), Paul Mitchell (*n* = 17), Robter D Frisina (*n* = 14), and Bamini Gopinath (*n* = 14). The top 10 co-cited authors in the field are shown in Figure 3C. Figure 3D is an overlay visualization map for author co-authorship analysis. In this study, G.A. Gates (448 citations) was the most representative author from the University of Washington in the United States, whose value of centrality was 0.22, occupied an absolute central position, followed by Lin FR, Schukecht HF.

### Contributions of the top 10 countries and regions

**Figure 4:**
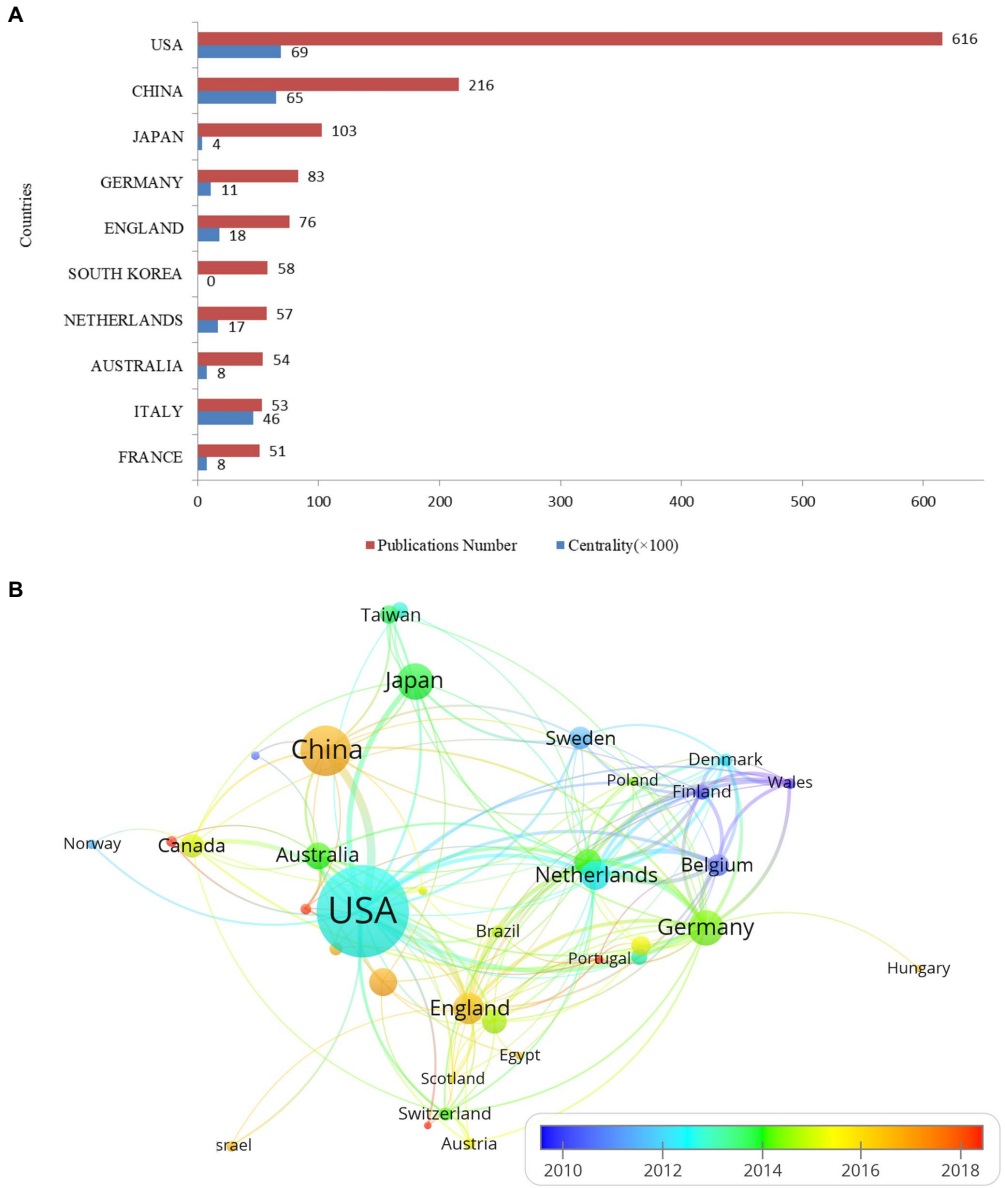
Top 10 productive countries on aged-related hearing loss research from 2002 to 2021 **(A)** and **(B)** cooperation network map of countries/regions. United States, United State of America.

During 2002–2021, a total of 62 countries and regions published articles on ARHL. As shown in Figure 4A, the United States published the most articles (*n* = 616), followed by China (*n* = 216), Japan (*n* = 103) and Germany (*n* = 83), showing that the countries mentioned above are in a leading position on this topic by using CiteSpace. Centrality is utilized to measure the likelihood of any shortest path through a node in the network maps (Wu et al., 2021a). The centrality of this nodes can reflect its importance within the networks. The United States (0.69) and China (0.65) have the top 2 centrality, indicates these two countries also have a dominant position in the field of ARHL.

Moreover, a country co-authorship overlay visualization map was generated by VOSviewer (Figure 4B). Each node represents a different country or region, and the node size is proportionate to the quantity of publications produced by that country. The distance between two nodes indicates the relatedness of their co-authorship link, and a smaller distance implies a stronger correlation of their relatedness. The thickness of the connecting line between nodes indicates link strength of a co-authorship relationship, which could be weighted by a quantitative indicator. The color of each node represents the average appearing year (AAY) for the country. The nodes given blue or green color indicate that most participants of these countries entered in the early stage of ARHL research, while the nodes given red color represent the new entrants of this field. Of the 38 countries/regions with a minimum number of 5 publications, used to construct the co-authorship network, the United States, China, Japan, Germany, and England play higher intermediary roles in the national cooperation network. In other countries, centrality and cooperation are relatively lower, which shows that most institutions have a low influence, and they have cooperated less with other countries.

### Article distribution among the leading institutions

**Figure 5:**
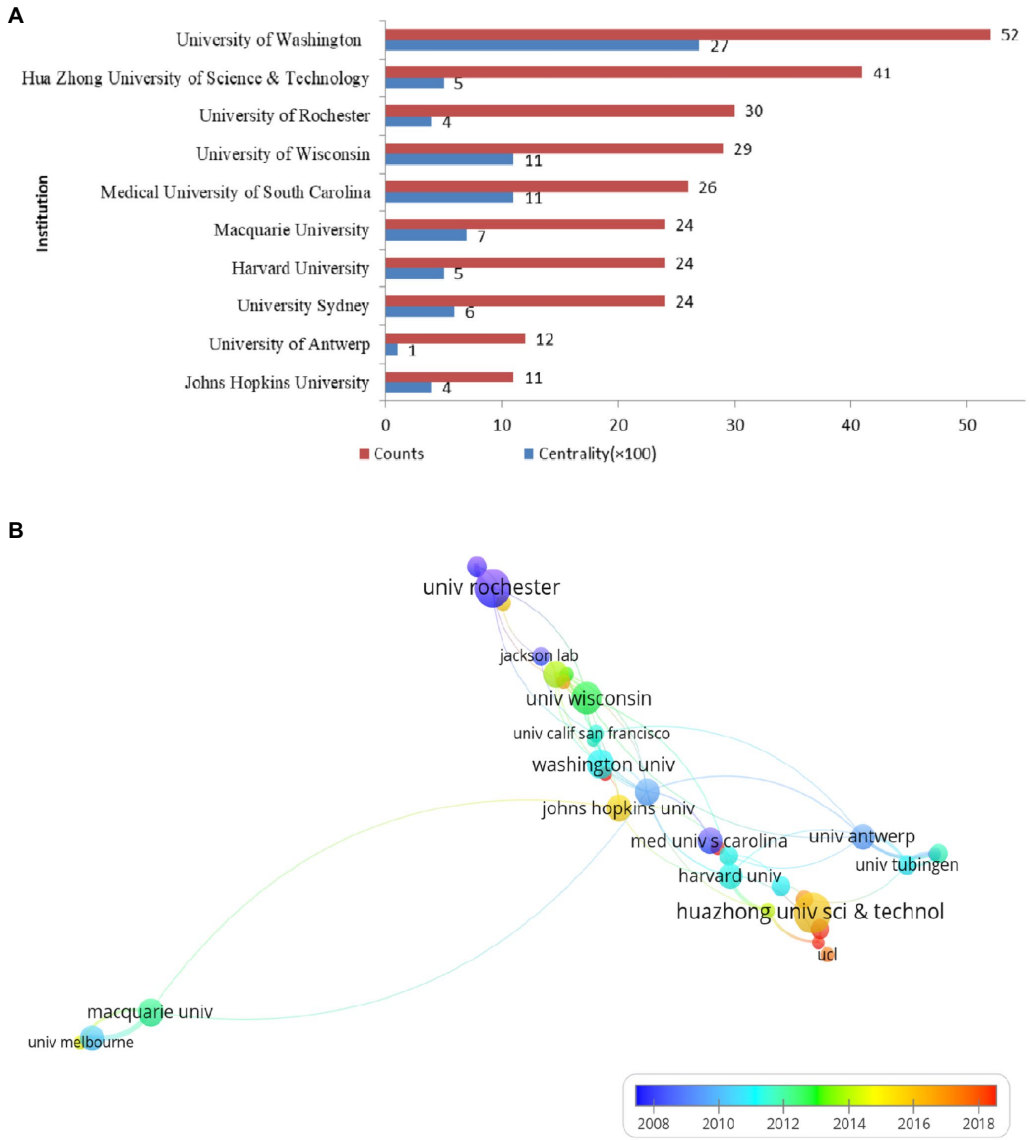
Top 10 institutions in terms of the number and centrality of studies published in 2002–2021 **(A)** and a network map of the institutions **(B)**.

In total, 1,499 institutions published articles on ARHL. Figure 5A shows the top 10 institutions that published literature on the field of ARHL from 2002 to 2021.The top 1 institution was University of Washington (*n* = 52) from the United States. The other top 5 institutions with high published literatures were Hua Zhong University of Science & Technology (*n* = 41), University of Rochester (*n* = 30), University of Wisconsin (*n* = 29), and Medical University of South Carolina (*n* = 26). University of Washington also had the biggest centrality (0.26), which indicates its central position in the field, followed by University of Wisconsin (0.11) and Medical University of South Carolina (0.11). The centrality of this institution was >0.1, indicating that it has a certain influence in the research field of ARHL. Figure 5B illustrates the cooperation network of the institutions by using VOSviewer. University of Antwerp (univ antwerp) (99), University of Oulu (univ oulu) (72) and University of Tubingen (univ tubingen) (71) are the top three institutions that have the high link strength, which means they have maintained intensive cooperation with others. The cooperation between other countries is relatively lower, and needs to be further strengthened.

### Article distribution among the leading journals

**Figure 6:**
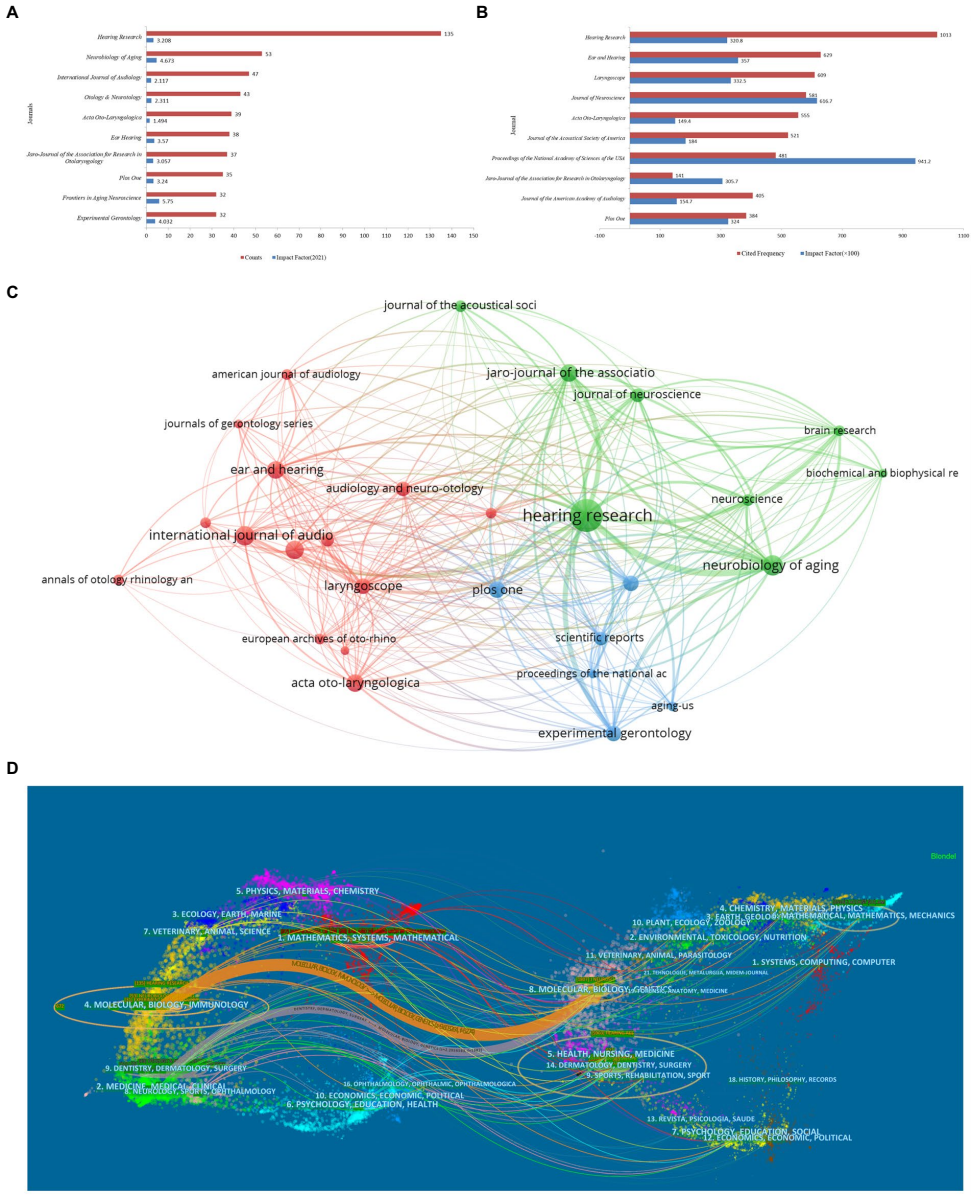
Top 10 leading journals related to aged-related hearing loss research from 2002 to 2021 **(A)**. Top 10 co-citation journals **(B)** and a network map **(C)**. Dual-map overlays of discipline and journal analysis **(D)**.

A total of 1,496 articles were published by 387 different journals in the field of ARHL. Figure 6A shows the top 10 journals in the last 20 years that published the most studies on ARHL. *Hearing Research* was well ahead of other journals with the largest number of articles (*n* = 136), followed by *Neurobology of aging* (*n* = 53), *International Journal of Audiology* (*n* = 47) (Figure 6B). Co-citation analysis of journals was performed by using the VOSviewer (Figure 6C). *Hearing Research* had the highest cooperation with other journals, followed by *Ear and Hearing*, *Laryngscope.* The highest impact factor journal was *Proceedings of the National Academy of Sciences of the* United States. Each article was labeled with one or more subject categories to facilitate rapid search in the WoSCC database. In this study, all the citations of journals can be clearly clustered into three categories: audiology or otolaryngology journals, the research direction of neuroscience and biological studies. Figure 6D shows the overlay of the journals and cited journals. We can see clearly how knowledge flows in different disciplines and identify the hotpot of each discipline. In the dual-map, the citing journals appear on the left side of the map, and the cited journals are on the right. The wider lines that begin from the citing journals and end at the cited journals represent the main citing pathways calculated from the so-called z-score of the citation links. The orange paths indicate that documents published in Molecular/Biology/Immunology journals usually cited documents published in journals belonging to Molecular/Biology/Genetics. The grey paths imply that the majority of papers published in the journals of Dentistry/Dermatology/Surgery are also likely to be biased to cite papers published in journals within Molecular/Biology/Genetics.

### Analysis of co-cited references

The top 10 references with the most citations in the field of ARHL are presented in Table 1. Among these articles, Gates and Mills (2005); Someya and Prolla (2010); Cosh et al. (2019) were the highest citated articles. The aforementioned paper published by Gates and Mills titled “Presbycusis,” had the highest impact factor (19.321) and was published in *Lancet*. Moreover, 50% of the studies published on the journals with impact factors were over 30, which mainly involved with inner ear and cochlear structure, and deafness genes, and prevention.

**Table 1:** Top 10 references on aged-related hearing loss.

Rank	Article	Count	Author/year	Journal	Impact factor (2021)
1	Sirt3 mediates reduction of oxidative damage and prevention of age-related hearing loss under caloric restriction	805	Someya et al. (2010)	*Cell*	41.582
2	Presbycusis	685	Gates and Mills (2005)	*Lancet*	79.321
3	The impact of hearing loss on quality of life in older adults	653	Cosh et al. (2019)	*Clinical Interventions in Aging*	4.458
4	Acceleration of age-related hearing loss by early noise exposure: evidence of a misspent youth	420	Kujawa and Liberman (2006)	*The Journal of Neuroscience*	6.617
5	Synaptopathy in the noise-exposed and aging cochlea: primary neural degeneration in acquired sensorineural hearing loss	374	Kujawa and Liberman (2015)	*Hearing Research*	3.208
6	Cadherin 23 is a component of the tip link in hair-cell stereocilia	323	Siemens et al. (2004)	*Nature*	49.962
7	Association of cadherin 23 with polygenic inheritance and genetic modification of sensorineural hearing loss	319	Noben-Trauth et al. (2003)	*Nature Genetics*	38.33
8	Hearing impairment and health-related quality of life: The Blue Mountains Hearing Study	305	Chia et al. (2007)	*Ear and Hearing*	3.57
9	Mammalian cochlear supporting cells can divide and trans-differentiate into hair cells	304	White et al. (2006)	*Nature*	49.962
10	SIRT3 protein deacetylates isocitrate dehydrogenase 2 and regulates mitochondrial redox status	278	Yu et al. (2012)	*The Journal of Biological Chemistry*	5.157

**Figure 7:**
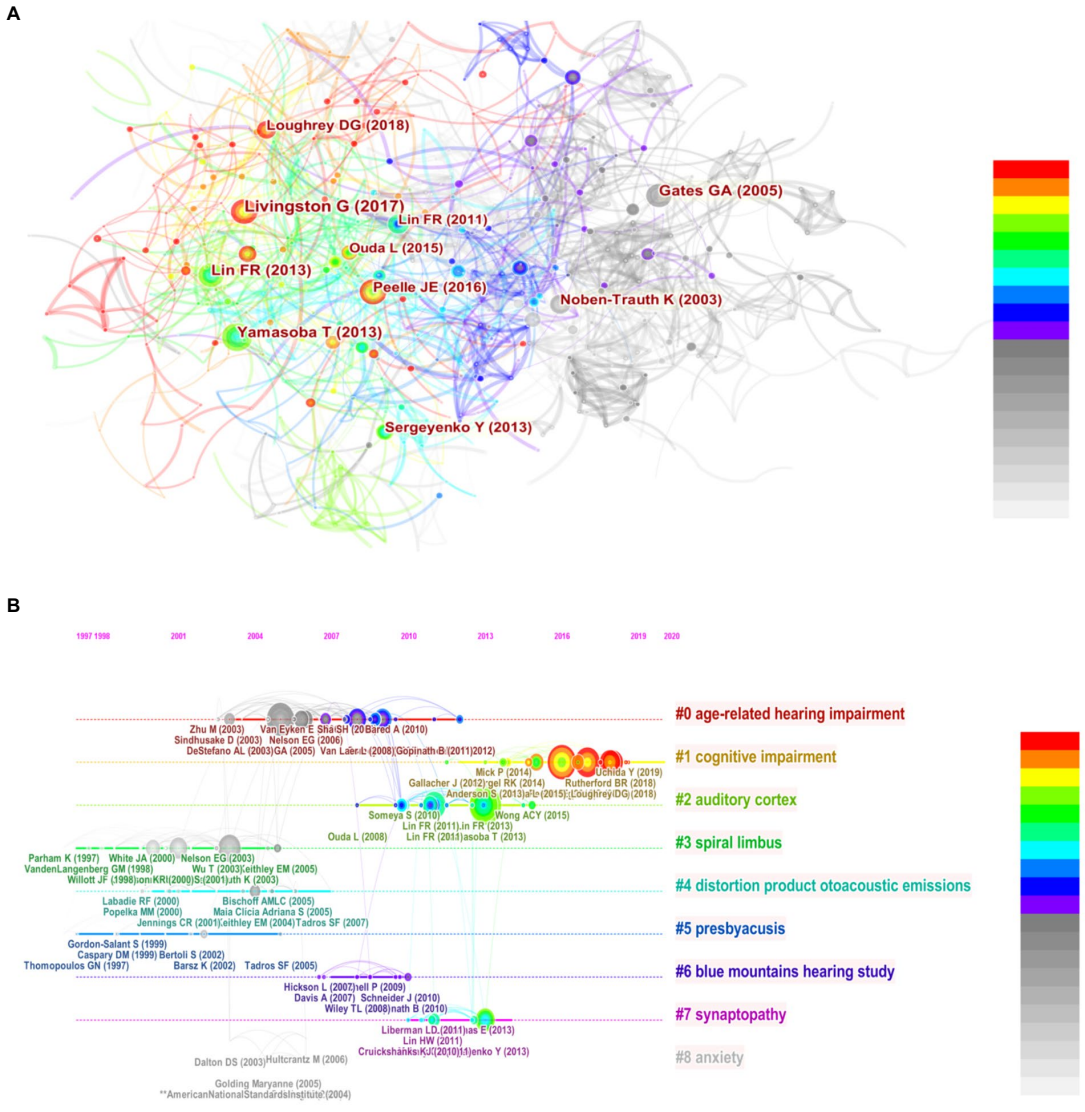
A network map showing the co-cited references **(A)** and the timeline view of co-cited clusters with cluster labels **(B)**.

Furthermore, co-cited references were analyzed by CiteSpace for visual correlation analysis (Figure 7A). References with citation bursts refer to those that have been frequently cited within one period of time. In this study, we set up the selection criteria to be the top 50 levels in a 1-year slice, and the minimum duration of the burst was set to 5 years. Each node represents a cited paper, and the red circles indicate a surge in citations. The top 3 authors were Peelle and Wingfiel (2016); Livingston et al. (2017); Loughrey et al. (2018), theirs papers have been more cited in recent 5 years, and played a leading role. The timeline perspective of the clustering diagram, combined with an analysis of clustering and time, displayed the distribution of topics in the field and the trends and the inter-relationships of research topics over time (Figure 7B). In this map, the blue lines indicated the time interval, and the red part represented the time period when the reference burst occurred. Among these references, the reference with the strongest burst value was written by Kleyer et al. In this study, the cluster theme is located in the rightmost diagram. The cluster analysis revealed eight clusters. The closest clusters on the timeline were “#0 age-related hearing impairment,” “#1 cognitive impairment,” and “#2 auditory cortex.” It is clearly seen that the keyword “#5 presbycusis” mainly appeared from 2007 to 2012, and replaced gradually by “#0 age-related hearing impairment.” The research related to “#8 anxiety” shown to be less after 2009, which was a research hotspot between 2002 and 2009.

### Keywords visualization

**Figure 8:**
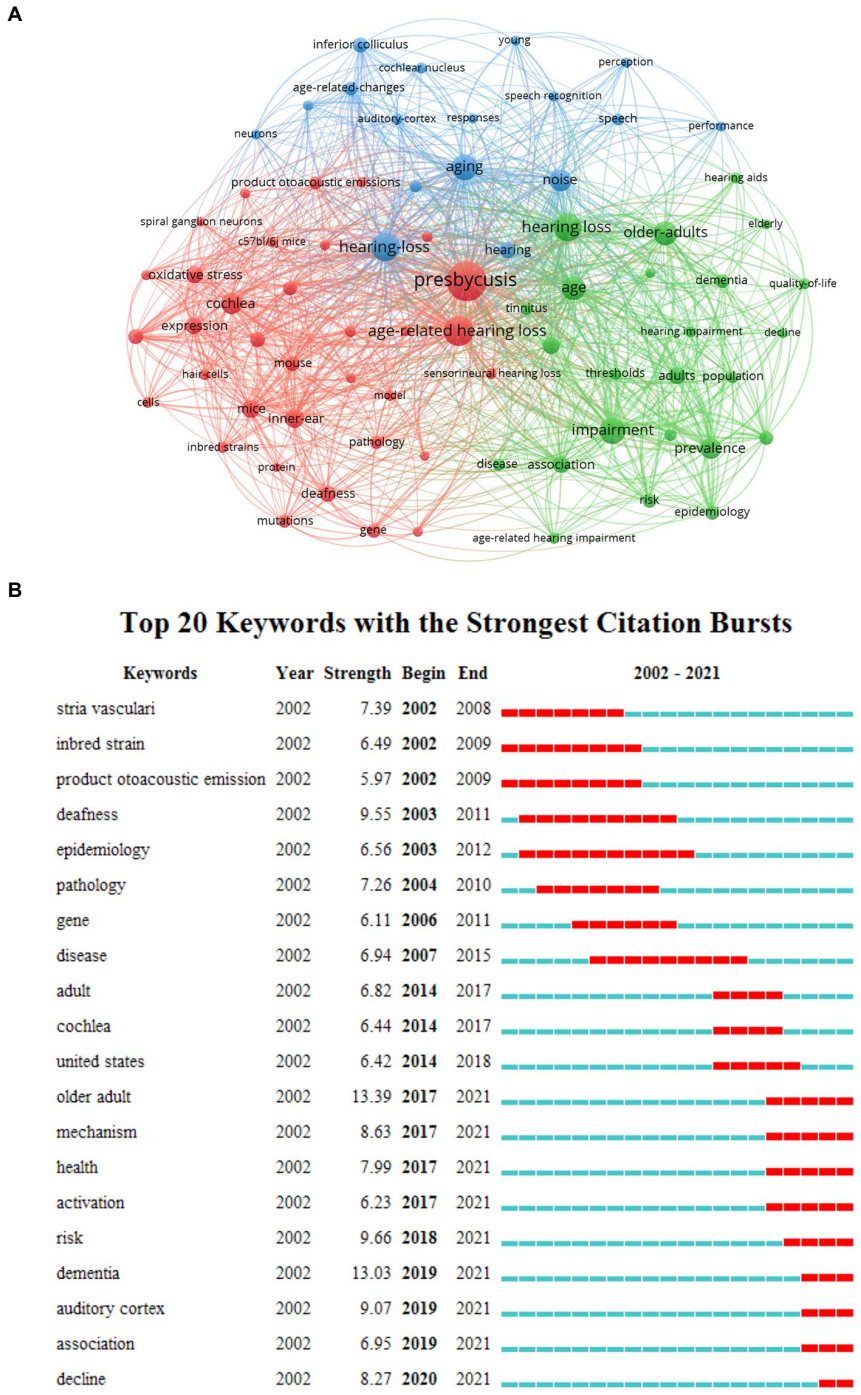
A network map of keywords in the research field **(A)** and keywords with strongest citation bursts **(B)**.

Figure 8A displays the keywords that occurred >30 times among 5,547 total keywords were analyzed using VOSviewer. The size of nodes is proportional to the occurrence times of keywords, and the relative distance between two nodes approximates the strength of their relationship. The thicker the lines between two nodes, the higher the frequency of their co-occurrence. Keywords with bigger circles in the density visualization suggested a research hotspot in this research area. The top 20 high-frequency keywords on aged-related hearing loss were shown in Table 2. These keywords can be divided into three clusters covering three aspects (1) laboratory research, pathology, animal models, and human genetics; (2) audiological characteristics and speech ability; (3) epidemiology and association, ARHL is considered as a risk factor for developing cognitive decline or dementia.

**Table 2:** Top 20 high-frequency keywords on aged-related hearing loss from 2002 to 2021.

Rank	Keywords	Count
1	Presbycusis	597
2	Hearing loss	519
3	Age-related hearing loss	295
4	Aging	220
5	Impairment	213
6	Age	181
7	Older-adults	178
8	Noise	153
9	Prevalence	148
10	Cochlea	137
11	Inner-ear	114
12	Mice	102
13	Oxidative stress	94
14	Adults	93
15	Association	93
16	Expression	91
17	Hearing	84
18	Deafness	83
19	Inferior colliculus	78
20	Stria vascularis	78

No, number.

The top 20 keywords with the strongest citation bursts in this field are presented in Figure 8B. The green line represents the time period from 2001 to 2021, and the interval of the burst keyword is shown by the red line. The keyword with the greatest burst strength in the past 20 years were “older adult” (13.39) and “dementia” (13.03). Other burst keywords used in the past 5 years including “risk” (2018–2021), “dementia,” “auditory cortex,” and “association” (2019–2021), and “decline” (2020–2021).

## Discussion

### Main findings

The study not only provided a historical prospect in the in the research field of ARHL, but also explore the research trends and hotspots. More than 5,514 authors published 1,496 articles on ARHL in 387 journals. The earliest publication was published in 1934 by M. Yearsley. Over the past 20 years, the overall volume of annually published research papers related to ARHL has increased with three fluctuations. However, there is an obvious upward trend. It is predicted that this research field may remain a hotspot. Moreover, the top journals (such as *Lancet* and *Cell*) have specially published special columns on ARHL. Based on the analysis of the number of citations published, the most frequent citation (805) by Someya and Prolla was published in *Cell* (Gates and Mills, 2005).

### Productive authors, countries, institutions, and journals

Weijia Kong from China published the most papers during recent 20 years. The top 10 most productive authors come from different countries, and they are distinguished professors in the field of ARHL. After e analysis of the data of authors, we found that they have a weak links and collaborative relationship relatively. The basic research of Audiology or otology is the main research interests of most of them in common. The author co-citation analysis is often used to reveal the key authors in a co-citation network of a particular field. Generally, frequently cited authors are thought to have a greater influence than those less cited. And authors who are jointly cited are likely to focus on similar research areas (Wu et al., 2021c). Among co-cited authors, G.A. Gates, Lin FR, Schuknecht, Ohlemiller K.K., and Willott JF contributed to more publications to advancing the global exchange and collaboration in this research field, and can be regarded as the leaders over the last 2 decades.

The United States (45.06%), China (15.80%), Japan (7.53%), Germany (6.07%), and England (5.56%) ranked the top five productive countries, accounting for 80.02% of the total publications. 618 articles were published from the United States, showed its tremendous impact on this field in publication outputs, total citations, and international collaborations. Compared with the continuously high proportion of the United States, Australia and some countries in Europe, publications from China, Japan, and South Korea have increased significantly, and they also have high centrality in the recent decade. This growth trend shows that countries in Asia are developing rapidly in this research field and are gradually developing towards high-quality research. Research institutions, such as the University of Washington from the United States, University of Wisconsin from the United States, Hua Zhong University of Science and Technology from China, contributed to high centrality. However, institutions show much lower cooperation than countries, this suggests that further international cooperation between institutions needs to be strengthened.

The presentation of research results in an international peer-reviewed journal is an essential component to establish effective scientific communication. The analysis of the distribution of journal sources is helpful for researchers to quickly find the most appropriate journals for their articles (Wu et al., 2021c). After the cluster analyzing of the journals, one-third of the top 10 journals refer to otolaryngology and audiology journals, the aims of these journals are concerned with basic peripheral and central auditory mechanisms, especially auditory anatomy, physiology, psychophysics, imaging, modeling and behavioral studies in animals and humans, as well as hearing aids and cochlear implants. The rest were classified as interdisciplinary journals, such as neurobiology, molecular biology and gerontology. This result shows that other disciplines are also paying closely attention to ARHL, and strengthen cooperation and exchange between different subjects in the future. Meanwhile, an analysis of the overlay of the journals and cited journals manifests basic medicine and clinical medicine interact closely.

### Co-cited references analysis

The analysis of reference co-citation analysis was a valuable technique to assess the evolution and trace the developmental frontiers of any research field. It is generally believed that the number of citations could reflect the impact extent of a publication, and the higher citations frequency indicates a higher academic level in a field (Wu et al., 2021c). A total of top 10 co-cited references from 2002 to 2021 show that researchers are growing more focused in their research on laboratory research, pathology, animal models, and human genetics related to ARHL. Half of them were published by high score journals, such as *Cell*, *Nature*, and *Nature genetics*. In addition, the article “presbycusis” published by Gates and Mills (2005) is regarded as an important and classic review in the research field of ARHL, whose impact factor is 79.321. This paper comprehensively reviewed the structure and function of the ear, the altered functions in presbycusis, diagnostic considerations, and treatment options. However, the keyword “presbycusis” ranked first in research frontiers and appeared earlier, and the keywords “age-related hearing loss,” after 2012. Our analysis of the co-cited literatures also contain the impact of hearing loss on quality of life in older adults, which has gained increasing research interest in recent years. ARHL can lead to communication difficulties with a perceived reduction in quality of life (Cosh et al., 2019). It can be attributed to the life expectancy increases and older adults are living longer, people with ARHL will be forced to endure hearing loss during their senior years. Therefore, more and more references pay attention to this aspect of research related to ARHL.

### Keywords and burst keywords analysis

Burst keywords are important indicators of emerging trends and research frontiers. Burst detection, an algorithm developed by Kleinberg, was an effective analytic tool to capture the sharp increases of references or keywords popularity within a specified period. This function can serve as an efficient way to identify concepts or topics that were actively discussed during some period of time (Wu et al., 2021b). Based on this, CiteSpace was used to identify burst keywords, and it seems that “mechanism,” “association,” “dementia,” “risk,” and “decline” have been the strongest burst keyword since 2017. A large number of studies have found that the hearing loss predated and predicted a subsequent clinical diagnosis of dementia (Thomson et al., 2017; Zheng et al., 2017; Loughrey et al., 2018; Liang et al., 2021). Dementia or cognitive decline is a devastating disease and global health challenge that is highly prevalent worldwide. The most widely proposed model suggested that the lack of auditory input and subsequent social isolation induced a preclinical cognitive decline (Chern and Golub, 2019). More research has shown an independent association between ARHL and dementia, identifying ARHL as a compelling potential target in preventive strategies for dementia. A causal linkage between ARHL and dementia needs to be investigated before making definitive clinical guidelines and treatment recommendations regarding ARHL as a modifiable risk factor. As medical evaluation technology, such as auditory function assessment, imaging examination, and central system examination, is developed in the future, progress in research about the association of ARHL with cognitive decline will be anticipated (Jafari et al., 2021). Although ARHL cannot endanger life, it is associated with social isolation, frailty, depression, and decreases in the quality of life. Therefore, how to further validate those associations and treat them may be the next research trend.

Nowadays, the research direction of laboratory research related to age-related hearing loss revealed a shift from microstructure (“oxidative stress” and “stria vascularis”) and neurology or brain (“inferior colliculus”) to the molecular mechanism (“mechanism”). The mechanism of hearing loss caused by aging need to be further clarified in the future, so then it can provide more effective treatment and Interventions. Furthermore, the results revealed that the keywords “older adult” and “health” began to appear in the past 5 years. Epidemiological investigation is the lasting research hotpot in this research field. The United States (Gopinath et al., 2021), China (Chen and Yu, 2020), and other regional or global investigations (Roth et al., 2011; Chadha et al., 2021) have conducted standardized large-scale epidemiological investigations since the past 5 years. The world has already began to pay attention to ear and hearing health, with the hope to relieve the risk in the field of hearing health rehabilitation through cost-effective clinical and public health solutions (Brown et al., 2018). The World Report on Hearing was developed with the purpose of global action to integrate ear and hearing care into a national health system framework (Chadha et al., 2021).

## Strengths and limitations

We chose to use the WoSCC database, because it is informally considered as the most accurate bibliographic source in the world, and has a strict assessment of publications and guarantees the high quality of the literature. Moreover, the WoSCC database spans over 100 years, and includes several dozens of millions of publication records. Therefore, the WoSCC database is used in bibliometric analysis widely. Further, we used the latest screening principles.

Nevertheless, a general limitation of our analysis is that should be considered. Firstly, we only chose Web of Science to search the publications, and may lead to incomplete literature retrieval. Secondly, we only included the English publications in our analysis. Lastly, the possibility of a certain bias in the selection of publications should not be excluded. Regardless of these limitations, we still revealed the future research trends and hotspots in ARHL to some extent.

## Conclusion

In conclusion, the annual number of publications has grown rapidly in the past two decades and will continue to grow. The United States is the leading country in this research field. China and Japan also reported some important research results and play a certain role in promoting the development of this research field. Epidemiological investigation and laboratory research are lasting hot spots, besides future research will focus on the association between ARHL and cognitive decline, dementia, and Alzheimer’s disease. The results herein provide a new perspective on the study of ARHL.

## Data availability statement

The original contributions presented in the study are included in the article/supplementary material, further inquiries can be directed to the corresponding authors.

## Author contributions

QC and NC designed this study, collected and analyzed the data, and drafted the manuscript. QC, NC, CW, and LH interpreted and critically assessed the results. JX and LH further revised the manuscript. All authors contributed to the article and approved the submitted version.

## Funding

This work was funded by the Science and Technology Development Program of the Beijing Rehabilitation Hospital (2020–043).

## Conflict of interest

The authors declare that the research was conducted in the absence of any commercial or financial relationships that could be construed as a potential conflict of interest.

## Publisher’s note

All claims expressed in this article are solely those of the authors and do not necessarily represent those of their affiliated organizations, or those of the publisher, the editors and the reviewers. Any product that may be evaluated in this article, or claim that may be made by its manufacturer, is not guaranteed or endorsed by the publisher.
